# An mRNA-based workflow validating neo-epitope presentation through HLA-I/peptide affinity purification

**DOI:** 10.3389/fimmu.2025.1566461

**Published:** 2025-06-04

**Authors:** Arthur Esprit, Dorien Autaers, Kris Thielemans, Elise Pepermans, Kurt Boonen, Geert Baggerman, Lorenzo Franceschini, Karine Breckpot

**Affiliations:** ^1^ Translational Oncology Research Centre, Vrije Universiteit Brussel (VUB), Brussels, Belgium; ^2^ Centre for Proteomics, Universiteit Antwerpen, Antwerp, Belgium; ^3^ ImmuneSpec BV, Niel, Belgium

**Keywords:** IVT-mRNA, epitope, presentation, purification, mass spectrometry

## Abstract

Presentation of human leukocyte antigen (HLA)-class I-restricted neo-epitopes is key for inducing an adaptive cytotoxic T-lymphocyte response against cancer. Validating presentation of these cancer-specific neo-epitopes following delivery to antigen-presenting cells (APCs) is critical to advance personalized therapeutic cancer vaccines. Current workflows for neo-epitope identification are often laborious and depend on computational deconvolution to determine the correct peptide sequence and its corresponding restriction element. We evaluated an mRNA-based workflow for more precise purification of HLA-I-peptide (pHLA)-complexes, facilitating peptide identification by liquid chromatography-tandem mass spectrometry (LC-MS/MS). This approach uses mRNA encoding a specific HLA-I-molecule fused to a Twin-Strep-Tag (HLA-TST), allowing affinity-based purification and downstream analysis of pHLA-complexes. As a proof-of-concept, we co-electroporated mRNA encoding TST-HLA-A*02:01 and mRNA encoding an HLA-A*02:01-restricted epitope in HLA-A*02:01-negative APCs. We demonstrated successful purification and detection of the delivered epitope via LC-MS/MS. These findings highlight the potential of the mRNA-based workflow to verify neo-epitope presentation by APCs. Still, further investigation is necessary to fully understand the technical variables that can influence peptide identification by LC-MS/MS.

## Graphical Abstract

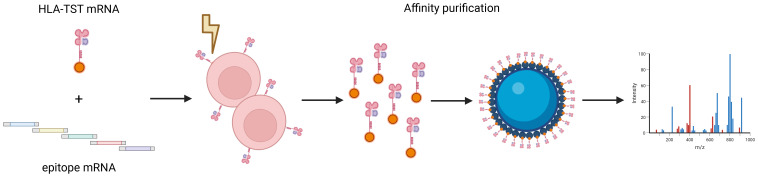

## Introduction

CD8^+^ T cells have the potential to eliminate cancer cells following recognition of cancer-specific and cancer-associated epitopes presented in human leukocyte antigen class I (HLA-I)-molecules on the cell surface of cancer cells (1, 2).

HLA-I-restricted epitopes (peptides) are derived from cellular proteins of which cytosolic and nuclear proteins that are mainly degraded by the ubiquitin proteasome system (3). The proteasome generates oligopeptides with a 2–20 amino acid length of which the majority are rapidly hydrolyzed by cytosolic peptidases (4). Some of the oligopeptides are transported by the transporter associated to antigen presentation (TAP) to the endoplasmic reticulum (ER) where they are trimmed by aminopeptidases (APs) such as ERAP1 (5) to fit the closed binding groove of the HLA-I-molecule (6). The HLA-I-peptide (pHLA-I)-complex is then transported to the cell surface for inspection by the T-cell receptor (TCR) of CD8^+^ T cells (7). Both defective ribosomal products or DRiPs, newly synthesized proteins that are defective in sequence or folding (8), and well-folded more stable proteins are a source of HLA-I-presented epitopes (9).

The majority of presented cytosolic and nuclear epitopes consists of self-epitopes in healthy cells. Cancer cells accumulate genetic mistakes, resulting in expression of neoantigens and presentation of neo-epitopes that potentially can be recognized by CD8^+^ T cells (5). This understanding has driven innovations in cancer vaccination and TCR-based adoptive cell therapies, thereby increasing interest in identifying cancer-specific neo-epitopes with high immunogenicity for therapeutic applications (10–12). Neo-epitope immunogenicity is particularly important for personalized vaccine development. Current immunogenicity studies have demonstrated that some neo-epitopes elicit strong T-cell responses, while others do not activate T cells at all (13). However, a caveat in these studies, is the lack of information on neo-epitope presentation, which could be interrogated by mass spectrometry (MS)-based approaches.

MS is widely employed to validate neo-epitope presentation, allowing identification of thousands of purified immunopeptides from a single sample (14, 15). Despite significant advances in MS-sensitivity, a critical factor in neo-epitope identification and validation is the sample preparation protocol. Ideally, the starting material should consist of a large number of highly HLA^+^-cells to ensure sufficient peptide yield for reliable identification (11). The first step in sample preparation involves enrichment of pHLA-complexes, typically by immunoprecipitation on a solid phase, such as a protein-A column, followed by acidic elution of peptides from the HLA-binding groove. The diversity of peptides identified is influenced by practical aspects of this workflow, necessitating thorough optimization to maximize the detection of relevant peptides (16). A consideration in this workflow is that current sample preparation protocols often depend on the W6/32 monoclonal antibody to enrich pHLA-I complexes (17, 18). However, this antibody does not discriminate between different HLA-I-molecules, resulting in a pool of epitopes that must be deconvoluted to their respective HLA-I-allotype. Additionally, allotype-specific anti-HLA-I-antibodies are not available for most HLA-allotypes. We addressed this limitation by designing fusion proteins, consisting of HLA-I-molecules fused to a Twin-Strep-Tag (HLA-TST), for affinity-based purification and downstream analysis of pHLA-I-complexes by liquid chromatography-tandem MS (LC-MS/MS). HLA-TST mRNA was co-electroporated with mRNA encoding a relevant epitope in antigen-presenting cells (APCs), demonstrating successful purification and detection of the delivered epitope via LC-MS/MS. These proof-of-concept findings highlight the potential of the mRNA-based workflow to verify neo-epitope presentation by APCs, while the same APCs can in principle be used to stimulate T cells for immunogenicity assessment, providing an integrated approach.

## Results

### An mRNA-based strategy for isolating pHLA-I-complexes for LC-MS/MS-analysis

To selectively enrich HLA-I-molecules and their associated peptides for LC-MS/MS-analysis, we used StrepTactin-based affinity purification, established for enriching recombinant proteins (19). The TST-coding sequence was fused *in silico* to the C-terminus of the HLA-A*02:01 α-chain gene and cloned in the pLMCT-plasmid for production of *in vitro* transcribed HLA-TST-mRNA.

Once presented, the pHLA-I-complex represents a molecular assembly composed of an 8–15 amino acid-long peptide, a polymorphic heavy chain and a covalently linked invariable β2-microglobulin (β2m), which acts as a chaperone to stabilize both empty and peptide-bound HLA-I-molecules. Given this structural complexity, and to ensure rigor in our mRNA-based approach for expressing TST-fusion proteins suitable for affinity capture, we also produced an additional mRNA-construct, encoding the TST fused to blue fluorescent protein (BFP). To that end, the BFP gene was fused to the TST-coding sequence at its C-terminus within the pLMCT-plasmid for subsequent *in vitro* transcription.

**Figure 1:**
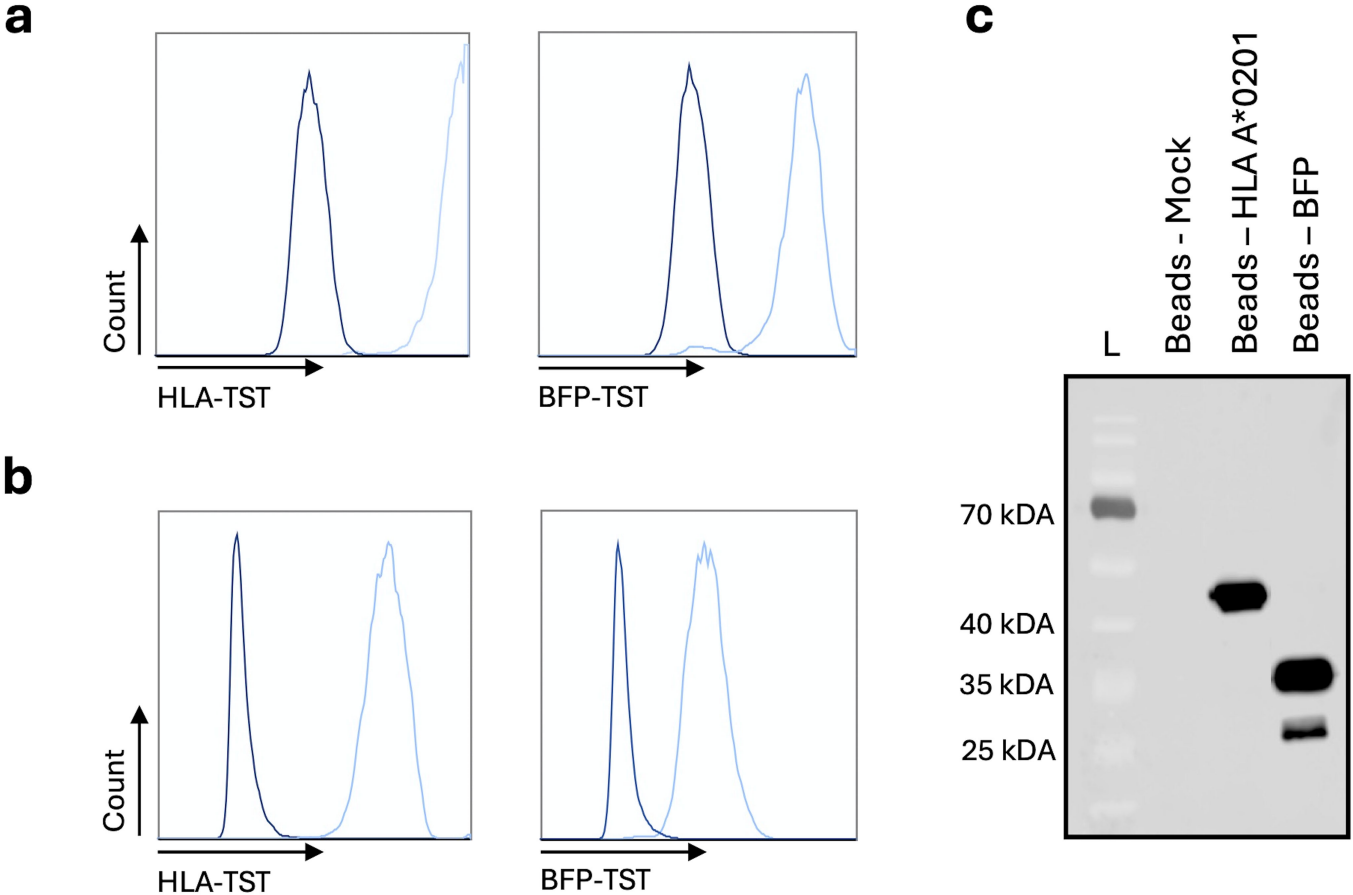
StrepTactin-based affinity capture of HLA-A*02:01-TST or BFP-TST in lysates of HLA-A*02:01-negative K562-cells electroporated with HLA-A*02:01-TST or BFP-TST-mRNA. Histograms show HLA-A*02:01 (left) or BFP (right) on **(a)** mRNA (light blue) or Mock (dark blue) electroporated cells, or **(b)** StrepTactin beads after affinity capture. **(c)** Western blot showing HLA-A*02:01 and BFP on StrepTactin beads after affinity capture. The data are representative for 3 independent experiments (n=3).

The BFP-TST-mRNA or HLA-A*02:01-TST-mRNA was electroporated into HLA-A*02:01-negative K562-cells, representing model APCs. HLA-A*02:01-negative K562-cells electroporated without mRNA (Mock) were used as a negative control. Expression of BFP-TST and HLA-A*02:01-TST following mRNA electroporation was demonstrated in flow cytometry (**Figure 1a**). Enrichment of BFP-TST and HLA-A*02:01-TST from cell lysates was achieved by StrepTactin-based affinity capture, as demonstrated in flow cytometry (**Figure 1b**) and western blot (**Figure 1c**).

**Figure 2:**
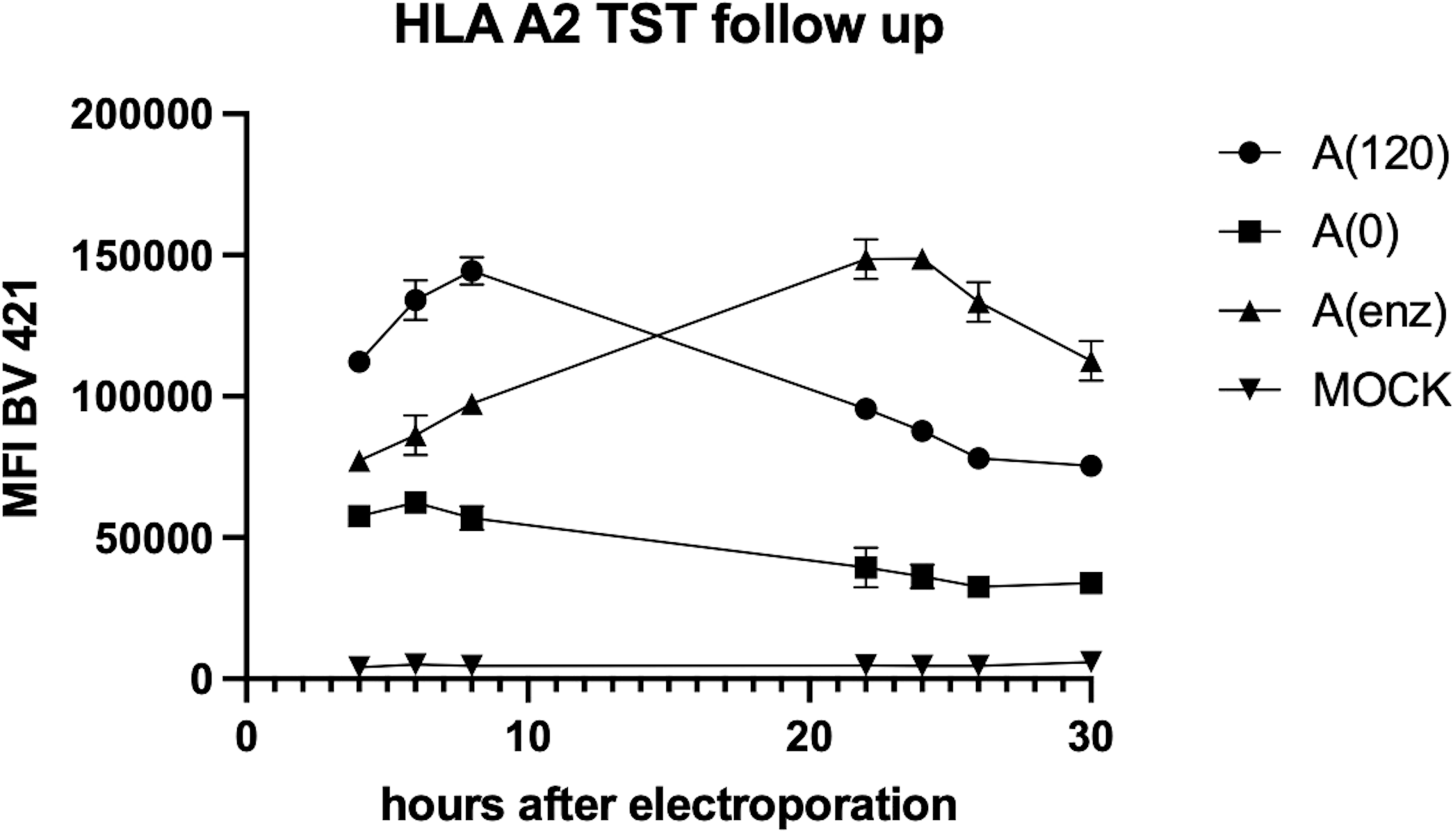
Poly-A-tailing strategies of *in vitro* transcribed HLA-A*02:01-TST-mRNA impact HLA-A*02:01-TST-expression kinetics. XY graph showing HLA-A*02:01-TST-expression over time after electroporation of HLA-A*02:01-negative K562-cells with differently poly-A tailed HLA-A*02:01-TST-mRNA. The XY graph summarizes the results of 3 independent experiments as mean ± standard deviation (SD).

We studied poly-A-tailing strategies to optimize the design of HLA-TST-mRNA and ensure high pHLA I-expression, as the poly-A-tail is a key determinant of mRNA-stability and translation efficiency (20). We compared co-transcriptional tailing with 120 adenosine residues [A(120)] to enzymatic tailing [A(enz)]. Expression of HLA-A*02:01-TST following mRNA-electroporation into HLA-A*02:01-negative K562-cells was monitored for 30 hours using flow cytometry and western blot. Higher HLA-A*02:01-TST-expression was observed following electroporation of A(120) or A(enz) HLA-A*02:01-TST-mRNA compared to Mock-electroporated HLA-A*02:01-negative K562-cells and HLA-A*02:01-negative K562-cells electroporated with mRNA lacking a poly-A-tail [A(0)]. Notably, HLA-A*02:01-TST-expression kinetics were distinct when using A(120) or A(enz) HLA-A*02:01-TST-mRNA with peak expression at 8 and 24 hours after electroporation respectively (**Figure 2**). This was corroborated by western blot (**Supplementary Figure S2**).

**Figure 3:**
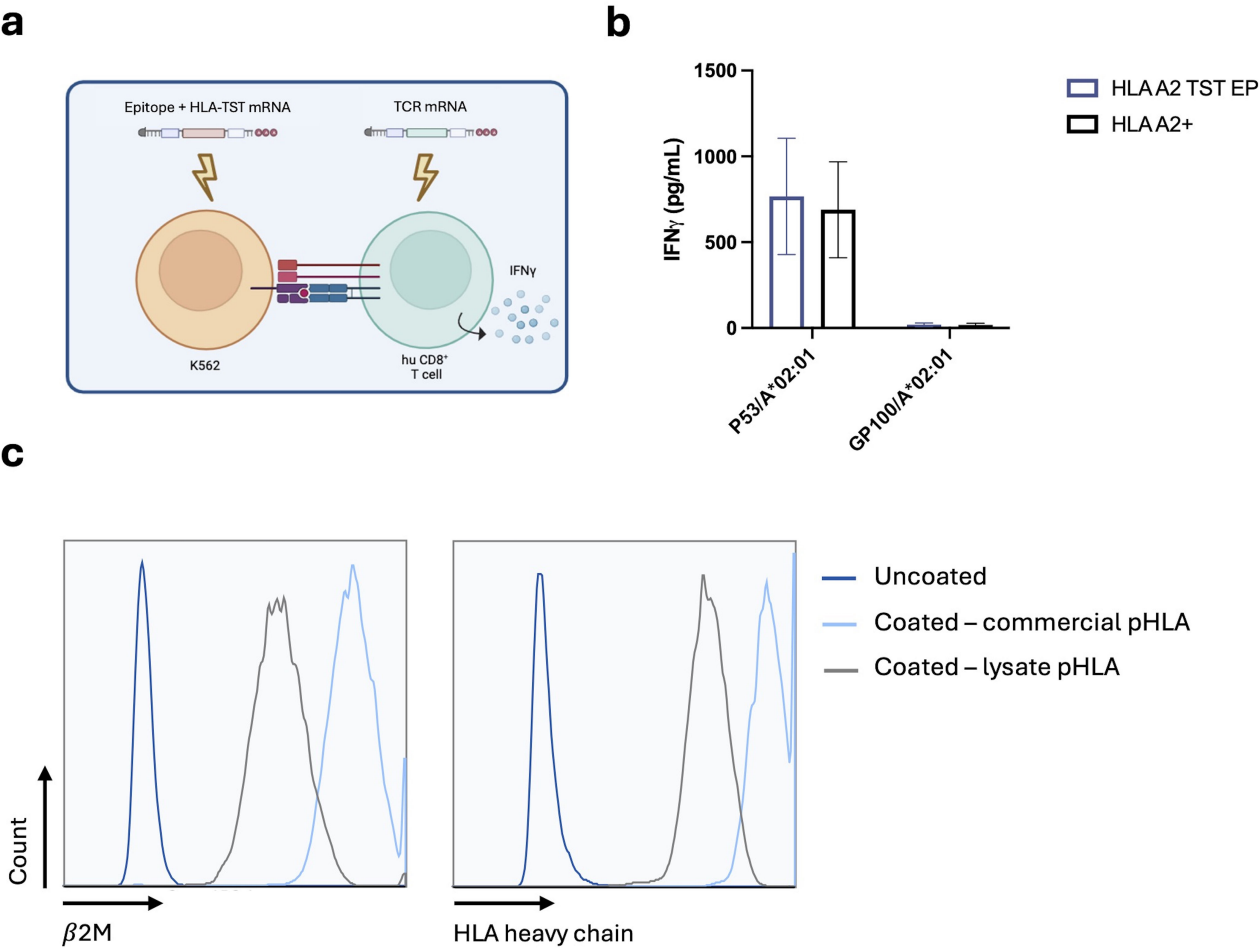
Structural validation of the pHLA-TST-complex following delivery of mRNA encoding HLA-A*02:01-TST and antigen. **(a)** Schematic representation of the antigen presentation assay. **(b)** Graph showing the production of IFN-γ by p53-specific T cells following co-culture with HLA-A*02:01-positive K562-cells (black bar) and HLA-A*02:01-negative K562-cells modified to express HLA-A*02:01-TST (blue bar), both electroporated with p53-mRNA or gp100-mRNA, as indicated in the x-axis. The graph summarizes data of 3 independent experiments as mean ± standard error of the mean (SEM) (n=3). Statistical significance was determined using multiple unpaired t-tests. **(c)** Histogram showing the expression of β2m (right panel) or HLA-A*02:01 (left panel) on uncoated StrepTactin beads (dark blue line), StrepTactin beads coated with in-house generated lysates from p53 and HLA-A*02:01-TST-mRNA electroporated HLA-A*02:01-negative K562-cells (black line) or coated with commercially available recombinant pHLA-A*02:01-TST-complexes (light blue line). The data are representative for 3 independent experiments (n=3).

We next addressed whether the incorporation of the TST at the C-terminus of the HLA-molecule allows generating pHLA-I-complexes. We co-electroporated HLA-A*02:01-negative K562-cells with mRNA encoding HLA-A*02:01-TST and mRNA encoding the HLA-A*02:01-restricted p53-epitope LLGRNSFEV. HLA-A*02:01-negative K562-cells co-electroporated with mRNA encoding HLA-A*02:01-TST and mRNA encoding the HLA-A*02:01-restricted gp100-epitope YLEPGPVTA were used as a negative control, while HLA-A*02:01-positive K562-cells electroporated with mRNA encoding the HLA-A*02:01-restricted p53-epitope LLGRNSFEV served as a positive control. An overnight co-culture was performed with human CD8^+^ T cells isolated from peripheral blood mononucleated cells (PBMCs) of healthy donors, electroporated with mRNA encoding the p53/HLA-A*02:01-specific TCRα and TCRβ-chain (**Figure 3a**). Antigen-specific T-cell activation, measured by interferon (IFN)-γ in ELISA, was observed in response to p53-presentation by both HLA-A*02:01-positive and HLA-A*02:01-TST-mRNA electroporated K562-cells, with no significant differences between these conditions (**Figure 3b**).

We next captured pHLA-A*02:01-TST-complexes from cell lysates on StrepTactin beads and benchmarked these to StrepTactin beads coated with commercially available pHLA-A*02:01-TST. Flow cytometry analyses demonstrated the presence of both the HLA-A*02:01-heavy chain and β2m in pHLA-A*02:01-TST-complexes generated from the lysate, though at lower levels compared to the StrepTactin beads prepared with the store-bought pHLA-A*02:01-TST (**Figure 3c**).

### The mRNA-based HLA-A*02:01-TST-approach enables epitope detection via LC-MS/MS

**Figure 4:**
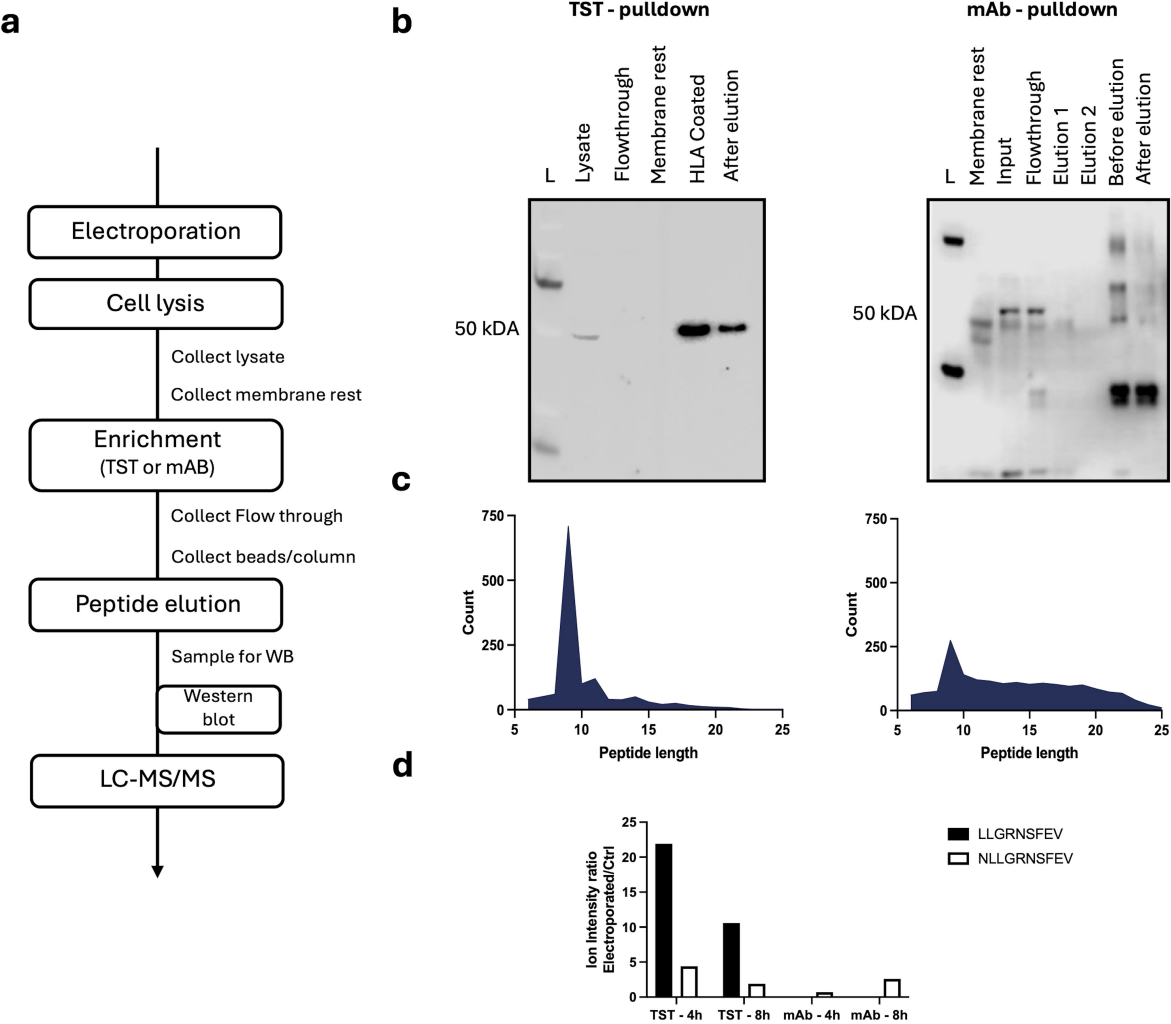
Applicability of the mRNA-based HLA-A*02:01-TST-approach for detection of an HLA-A*02:01-restricted epitope via LC-MS/MS. **(a)** Schematic representation of the key steps in the experimental protocol. **(b)** Western blot of samples collected during the enrichment workflow for both the TST-based (left) or antibody-based (right) approach. **(c)** Size distribution of peptides recovered from pHLA-A*02:01-complexes enriched using the TST-based (left) or antibody-based (right) approach. **(d)**. Bar chart showing the ion intensity ratio for the peptides NLLGRNSFEV and LLGRNSFEV purified with the TST- and antiboy (mAb)-approach performed at 4 hours or 8 hours after electroporation (n=1).

Having confirmed that HLA-A*02:01-TST, expressed on HLA-A*02:01-negative K562-cells following mRNA delivery, can be effectively captured on StrepTactin beads, and that the HLA-A*02:01-TST can present a mRNA co-delivered epitope to T cells, we next addressed if this approach enables epitope detection by LS-MS/MS. To validate efficient solubilization and depletion of pHLA-A*02:01-complexes, aliquots were collected at key stages of the experimental workflow for western blot and LC-MS/MS-analysis (**Figure 4a**). We enriched pHLA-A*02:01-complexes using antibodies or StrepTactin beads, starting with lysates of 100^E^6 HLA-A*02:01-negative K562-cells that were collected 4 or 8 hours after electroporation with HLA-A*02:01-TST-mRNA or HLA-A*02:01-mRNA, and p53-epitope mRNA. No detectable HLA-A*02:01-TST-signal remained in the membrane fraction following cell lysis, suggesting complete solubilization. StrepTactin-based affinity capture fully depleted pHLA-A*02:01-complexes from the lysate, whereas residual complexes were detectable following antibody-based capture on a protein-A-column (**Figure 4b**). Both methods yielded relatively small peptide pools, retrieving fewer than 1,000 unique peptides each. Notably, the peptide profiles differed between the two methods with primarily 9 amino acid-long peptides retrieved following StrepTactin-based enrichment and a larger number of 10 amino acid-long peptides retrieved following antibody-based enrichment (**Figure 4c**). Analysis of the peptide pools using FragPipe v21.1 showed the retrieval of two versions of the mRNA-delivered p53-epitope at both timepoints after electroporation (**Figure 4d**; **Supplementary Figure S3**). The 9-mer peptide LLGRNSFEV and 10-mer peptide NLLGRNSFEV were recovered with the TST-based approach, while only the 10-mer version was recovered with the antibody-based approach. These findings demonstrate the utility of the mRNA-based HLA-A*0201-TST-approach for enriching pHLA-A*02/01-complexes, facilitating epitope detection via LC-MS/MS.

## Discussion

Cancer immunotherapy increasingly focuses on mounting immunity against neo-epitopes expressed in a patients’ cancer. To identify these neo-epitopes, typical discovery workflows begin with NGS of patient cancer cells compared to healthy cells, followed by *in silico* prediction of tumor-specific neo-epitopes. This process results in an abundance of candidate neo-epitopes, necessitating neo-epitope prioritization to enable the development of personalized cancer immunotherapy strategies (21).

Epitope prioritization can be achieved by dry and wet lab approaches (22). One approach involves algorithm-based selection of neo-epitopes considering biophysical and chemical characteristics. However, algorithm prediction alone does not guarantee efficient presentation or immunogenicity, making wet lab validation key (22). Experimental validation of neo-epitope immunogenicity provides a feedback loop, allowing training and fine-tuning of the prediction algorithm. Ideally, cell-based assays studying neo-epitope immunogenicity should be combined with LC-MS/MS-validation of neo-epitope presentation to ensure that neo-epitopes are presented when identified as non-immunogenic (21).

We developed a workflow compatible with LC-MS/MS to enrich pHLA-A*02:01-complexes with HLA-allotype specificity. This approach can be applied to any HLA-allotype, ensuring that peptides identified by LC-MS/MS were enriched by the HLA-molecules. This avoids further deconvolution to match the correct epitope to the correct HLA-allotype, contrasting pHLA-enrichment using the W6/32 antibody that enriches pHLA-I-complexes in an allele-unspecific way. We used a TST-based approach, enabling StrepTactin-based affinity capture of pHLA-A*02:01-TST-complexes, while maintaining structural integrity, including epitope association. This fusion-protein was encoded in mRNA for efficient electroporation into APCs. Functionality was validated by co-electroporating the mRNA encoding HLA-A*02:01-TST with mRNA encoding an HLA-A*02:01-restricted p53-epitope, showing activation of p53-specific CD8^+^ T cells, efficient enrichment of pHLA-A*02:01-complexes by affinity capture on StrepTactin beads and identification of the co-delivered p53-epitope by LC-MS/MS.

Previous studies have explored viral vector-based methods to generate cell lines for specific pHLA-purification, though most did not consider a tag for affinity-based capture of the HLA-allotype (23). When a tag was used, it was based on a biotin acceptor peptide, which is 15 amino acids in length (24). In contrast, we used the TST, which is only 8 amino acids-long and offers the advantages of minimal interference with target protein structure or function, and micromolar affinity for StrepTactin. This allowed affinity-based capture and elution of TST-fusion proteins. Moreover, we chose to use mRNA for pHLA-A*02:01-TST-expression. mRNA has several advantages over viral vectors, most importantly the ease of manufacturing in standard laboratory setting, while further providing flexibility in experimental design. This adaptability contrasts the rigidity of antibody-based protocols, which require the development of allotype-specific antibodies, when used to enrich specific HLA-allotypes.

It is important to acknowledge that the total peptide yield in this study was low for both the TST-based and antibody-based enrichment methods, even though we started from a sample size of 100^E^6 APCs. This was unexpected as the sample size of the starting material was previously reported to be positively associated with the number of identified peptides (15). The low number of peptides identified is a limitation that might be due to the APCs and/or mRNA used in this study. We chose the human erythroleukemic K562-cells as model APCs, as these lack expression of HLA-alleles but do express β2m (25). As a result, introducing the HLA-A*02:01 α-chain into these cells suffices to acquire functional antigen-presenting pHLA-A*02:01-complexes on the cell surface. However, these APCs express constitutive proteasomes comparable to tumor cells (26), while professional APCs, typically targeted by cancer vaccines express immunoproteasomes that are more proficient at generating epitopes for pHLA-I-presentation (27). Therefore, using other APCs expressing immunoproteasomes or stimulating the expression of immunoproteasomes in K562-cells by pre-treating them with IFN-γ could offer a solution for this limitation. This hypothesis is supported by the study of Javitt et al., demonstrating a doubling in the number of recovered peptides when starting from 500^E^6 A549-cells that were treated with IFN-γ and tumor necrosis factor (TNF)-α (28). Also, the transient nature of HLA-A*02:01-TST and epitope expression following mRNA-delivery might contribute to the low recovery of peptides. We hypothesize that improving the mRNA-molecular design for high and durable protein expression in the targeted APCs and linked herewith determining the optimal timing for sample collection following mRNA-electroporation could improve the yield of recovered peptides. We showed that subtle differences in the mRNA-molecular design, in this study the poly-A-tail, impacts the protein expression kinetics, underscoring the need to determine the optimal mRNA-design and timing for sample preparation when using different mRNA-designs. Notably, any structure in the mRNA-molecule can impact protein expression (29).

Differences in peptide recovery were observed between the TST- and antibody-based enrichment methods, with more 10-mer peptides recovered with the antibody-based enrichment of pHLA-A*02:01-complexes, containing NLLGRNSFEV rather than the expected LLGRNSFEV, while both were identified with the TST-based method. The biological reason for this phenomenon remains to be explored. Notably, differences in peptide distribution following distinct methods of MS-sample preparation were also reported by others. It was demonstrated that epitope compositions vary significantly depending on the method used for pHLA-enrichment (30). When high-performance liquid chromatography (HPLC), C18-cartridges or 5 kDa filters were used, notable differences in peptide profiles were observed. HPLC-enrichment yielded more peptides with a proline at the anchor position P2, whereas C18-cartridges favored peptides with an arginine at this position. This highlights the influence of choice of pHLA-enrichment method on the diversity of recovered peptides and presents challenges when studying the endogenous immunopeptidome.

It should be acknowledged that LC-MS/MS-analysis is not free from errors, arising from technical limitations and bioinformatics challenges, in particular for the identification of singly charged immunopeptides, often with unideal ionizability characteristics. This analysis is facilitated by the prior knowledge of the targeted neo-epitope in the intended use, validation of neo-epitope presentation during immunogenicity screening. Still, there remains a risk of potentially missing information, as the exact number of peptides presented on cells is unknown and is often extrapolated from empirical data. These considerations highlight a need for further research on fundamental aspects of the immunopeptidome as well as the standardization of pHLA-enrichment methods and downstream analytical approaches.

In conclusion, we delivered a proof-of-concept for an mRNA-based approach for co-delivery of HLA-TST-molecules and neo-epitopes. This allows studying neo-epitope immunogenicity in T-cell assays, while performing targeted enrichment of HLA-I/neo-epitope complexes for validation of neo-epitope presentation by LC-MS/MS-analysis using simultaneously prepared APC-samples. As such, it can be excluded that lack of T-cell activation is due to faulty presentation of the neo-epitope. To fully exploit this promising workflow, further research is needed to optimize the process and account for technical variables that may impact peptide detection accuracy.

## Material and methods

### Cell culture and primary T cells

HLA-A*02:01-negative K562-cells were cultured as previously described (31). Leukapheresis was performed on healthy volunteers at the Hematology unit of the university hospital in Brussels (UZ Brussel) using the Spectra Optia^®^ apheresis system (Terumo BCT). The PBMCs were processed to separate monocytes and lymphocytes by counterflow cell centrifugation at the Translational Oncology Centre at the Vrije Universiteit Brussel (VUB, Brussels, Belgium). CD8^+^ T cells were further isolated from the lymphocyte fraction by MACS using positive selection with human anti-CD8 microbeads (Miltenyi Biotec).

### DNA synthesis

Plasmid DNA was generated using the in-house developed plasmid pLMCT. gBlocks coding for HLA-A*02:01-TST and a p53-cognate TCRα and TCRβ were purchased from Integrated DNA Technologies (IDT). These sequences were cloned into the pLMCT using the Gibson assembly kit™ (New England Biolabs, NEB) after restriction digestion with NcoI/XhoI (NEB). The resulting pLMCT-plasmids were transformed in XL2-Blue Ultracompetent Cells (Agilent) and selected on ampicillin containing agar plates. Cloned pLMCT-plasmids were screened based on the sequence specific restriction digestion pattern and were sequence verified (Eurofins Genomics). After amplification of selected bacterial clones, pLMCT-plasmids were isolated (Qiagen Midi Plasmid Kits) and linearized over night by restriction enzyme digestion with BfuAI (NEB). Synthetic DNA encoding the HLA-A*02:01-restricted LLGRNSFEV epitope of p53 or YLEPGPVTA epitope of gp100 were generated as described (13).

### *In vitro* mRNA transcription

The *in vitro* transcription reaction was performed using the linearized pLMCT-plasmid or synthetic DNA-template as previously described (13).

### mRNA electroporation

Electroporation was performed using the Gene Pulser X-Cell Electroporation System (Biorad) using 4 mm cuvettes. Cells were harvested and washed twice with OptiMEM before electroporation. For K562-cells, 2^E^6 cells were electroporated in a 200 μL OptiMEM-mRNA mix containing 5 μg of mRNA using an exponential decay protocol set at 300V, 125 μF capacitance and infinite ( ∞) Ω resistance. For electroporation of larger samples of 5^E^7 cells, the voltage was increased to 400V, and electroporation was performed in 800 µL containing a final concentration of 100 µg/mL mRNA (1:1 ratio of antigen and HLA mRNA). For CD8^+^ T cells, 4^E^6 cells were electroporated in a 200 µL OptiMEM-mRNA mix containing 5 μg TCRα and 5 μg TCRβ mRNA. A square wave protocol was applied, delivering a single 5 millisecond pulse at 500V.

### Flow cytometry

Cells were harvested and washed twice with phosphate-buffered saline (PBS) containing 1% bovine serum albumin (flow cytometry buffer). HLA-A*02:01-BV421 (BioLegend, clone BB7.2) was used at a 1:150 dilution. Viable cells were discriminated from dead cells using 7-amino actinomycin D (BD Biosciences). The gating strategy is shown in **Supplementary Figure S1**.

### T cell activation assay

For validation of antigen presentation, HLA-A*02:01-negative K562-cells were electroporated with mRNA encoding HLA-A*02:01-TST and mRNA encoding the HLA-A*02:01-restricted gp100-epitope YLEPGPVTA (negative control) or the HLA-A*02:01-restricted p53-epitope LLGRNSFEV. As a positive control, HLA-A*02:01-positive K562-cells were electroporated with mRNA encoding the HLA-A*02:01-restricted p53-epitope LLGRNSFEV. These cells were co-cultured overnight with human CD8^+^ T cells isolated from peripheral blood mononucleated cells (PBMCs) of healthy donors and electroporated with mRNA encoding the p53/HLA-A*02:01-specific TCRα and TCRβ-chain. Cells were co-cultured at a 1:1 ratio with 2.5^E^4 APCs in 200 μL of cell culture medium. After overnight incubation at 37°C, a human IFN-γ ELISA was performed according to manufacturer’s instructions (Invitrogen).

### Enrichment of pHLA-A*02:01-TST using StrepTactin bead-based precipitation

Electroporated cells were collected in a protein lo-bind tube (Eppendorf) and washed twice with PBS. The cell pellets were snap-frozen in liquid nitrogen. Thawed cell pellets were resuspended in cell lysis buffer (33 mL PBS, 400 µg CHAPS detergent, 1 tablet proteinase inhibitor) and incubated for 30-minutes at 4°C under constant agitation. Cell lysates were centrifuged at 800g for 5 minutes at 4°C to pellet the nuclei and cell debris. The supernatant was transferred and centrifuged at 16 000g for 15 minutes at 4°C to pellet the residual membrane fraction. Meanwhile, StrepTactin beads were washed twice with PBS before being spiked into the sample. After washing, 10 µL of StrepTactin beads were added to the lysate and incubated for 1 hour at 4°C under constant agitation. After incubation, bead-lysate mixtures were placed on a magnet for separation of the two components and beads were washed twice with PBS. StrepTactin beads, coated with pHLA-A*02:01-complexes, were further handled according to the application. Detection of pHLA-A*02:01 in western blot was performed with StrepTactin-HRP using a 1:10–000 dilution.

### Immunopeptidomics sample preparation

For purification of pHLA-A*02:01-TST-complexes using StrepTactin beads, 100^E^6 cells were lysed according to the protocol above in 4.5 mL lysis buffer and purified using 100 µL of StrepTactin beads. After binding, coated beads underwent consecutive washing with 500 µL of wash buffers 1 through 4 (WB1: PBS, 0,0012% (w/v) CHAPS, proteinase inhibitor, 0.5 mM EDTA; WB2: 150 mM NaCl, 50 mM Tris; WB3: 450 mM NaCl 50 mM Tris; WB4: 50 mM Tris). After washing, peptides were eluted from pHLA-A*02:01-TST-complexes using 10% acetic acid and purified by solid phase extraction using Seppak 1cc C18 columns (Waters). Briefly, after activation (3 CV’s 70% ACN, 0.1% TFA) and rinsing (3 CV’s 2% ACN, 0.1% TFA) the eluted fraction was loaded twice on the Seppak column. After 5 CVs of washing (2% ACN, 0.1% TFA) peptides were eluted in 35% ACN, 0.1% TFA prior to vacuum drying and storage in the ultra-freezer until LC-MS/MS-analysis. For the antibody-based enrichment of pHLA-A*02:01-complexes a lysate was prepared from 100^E^6 electroporated cells in 4.5 mL lysis buffer (150 mM NaCl, 50 mM Tris pH 8, 0.1% Igepal, supplemented with complete antiprotease inhibitor cocktail (Roche)) followed by affinity purification using antibody W6/32 crosslinked to protein-A-Sepharose beads (Cytiva). After binding, the beads were washed consecutively with following wash buffers (WB1: 150 mM NaCl, 50 mM Tris pH 8, 0.1% Igepal, 0.5 mM EDTA, supplemented with complete antiprotease inhibitor cocktail; WB2: 150 mM NaCl, 50 mM Tris pH 8, 0.1% Igepal, WB3: 450 mM NaCl, 50 mM Tris pH 8, 0.01% Igepal, WB4: 50 mM Tris). After washing, peptides were eluted from pHLA-A*02:01-complexes using 10% acetic acid and immunopeptides were purified by solid phase extraction as described above.

### LC-MS/MS analysis

Samples were dissolved in 10 µL of 2% acetonitrile (ACN) and 0.1% formic acid (FA) and separated on an ACQUITY UPLC M-Class System (Waters). The system was equipped with a nanoEase™ M/Z Symmetry C18 trap column (100 Å, 5 µm, 180 µm × 20 mm) and a nanoEase™ M/Z HSS C18 T3 analytical column (100 Å, 1.8 µm, 75 µm × 250 mm), both from Waters. Samples were loaded onto the trap column in 2 minutes at 5 µL/minute in 94% solvent A and 6% solvent B (solvent A is 0.1% FA in 18.2 MOhm∗cm water (MilliQ), solvent B 0.1% FA in 80% ACN). The flow over the main column was 0.4 µl/minute and the column was heated to 40°C after an isocratic flow of 4 minutes at 6% B. After an additional isocratic flow of 4 minutes at 94% B, the concentration of B decreased in 4 minutes to 6%, which was followed by 15 minutes of equilibration at 6%. The column was online with a timsTOF Pro operating in positive ion mode, coupled with a CaptiveSpray ion source (both from Bruker Daltonik GmbH, Bremen). The timsTOF Pro was calibrated according to the manufacturer’s guidelines. The temperature of the ion transfer capillary was 180°C. The Parallel Accumulation-Serial Fragmentation DDA method was used to select precursor ions for fragmentation with 1 TIMSMS scan and 10 PASEF MS/MS scans, as described by Meier et al. (32). The TIMS-MS survey scan was acquired between 0.70 and 1.45 Vs/cm^2^ and 100–1,700 m/z with a ramp time of 166 milliseconds. The 10 PASEF scans contained on average 12 MS/MS scans per PASEF scan with a collision energy of 10 eV. Precursors with 1–6 charges were selected with the target value set to 20,000 a.u and intensity threshold to 2,500 a.u. Precursors were dynamically excluded for 0.4 seconds. The timsTOF Pro was controlled by the OtofControl 5.1 software (Bruker Daltonik GmbH).

### Data analysis

Data were analyzed using FragPipe v21.1 with the inbuild Nonspecific-HLA workflow, which includes DB search and MSBooster-based rescoring, combined with IonQuant v1.10 for MS1 quantification (33). The reviewed human Uniprot proteome was used as a database (20482 entries, downloaded on March 25th, 2024) and Methionine oxidation and protein N-terminal acetylation were set as variable modifications. All other parameters were standard for the Nonspecific-HLA workflow. Lonquant settings included enabling “match between runs” and normalization of ion intensities by total peptide intensities. The database and parameters used for the Fragpipe data analysis workflow are available at the PRIDE repository (PXD062551). GraphPad Prism v9.5.0 was used for statistical analysis.

## Data Availability

The mass spectrometry proteomics data have been deposited to the ProteomeXchange Consortium via the PRIDE partner repository with the dataset identifier PXD062551 (34).

## Supplementary material

Supplementary Table 1 Overview of sequences.

Supplementary Figure 1 Flow cytometry gating strategies. Gating strategy for **(a)** K562-cells and **(b)** StrepTactin beads.

Supplementary Figure 2 Western blot showing HLA-A*02:01-TST-expression at 4, 8, 22 and 30 hours following electroporation of HLA-A*0201-negative K562-cells with HLA-A*02:01-TST-mRNA that lacked a poly-A-tail [A(0)] or that was poly-adenylated using an enzymatic [A(enz)]or co-transcriptional [A(120)] approach.

Supplementary Figure 3 Chromatographs of peptides recovered from pHLA-A*02:01-complexes enriched using the TST-based **(a)** or antibody-based **(b)** approach.
