# Supplementary material for: An mRNA-based workflow validating neo-epitope presentation through HLA-I/peptide affinity purification
Source: Front Immunol. 2025 Jun 4;16:1566461. doi: 10.3389/fimmu.2025.1566461 (PMC12174155; doi:10.3389/fimmu.2025.1566461)
Supplement: Supplementary Table 1 — Overview of sequences. [file Table1.pdf]

Table S1: Overview of sequences

Feature	DNA sequence
T7-promotor AG	taatacgactcactataag
5'UTR/KOZAK	gaatacaagctacttgttctttttgcatcacaaccaggcctccacaacc
3'UTR	ctcgagtgttttggctgggtttttccttgttcgcaccggacacctccagtgaccagacggcaaggtttttatcccagtgtatattgtcgac
HLA A*0201	atggctgtgatggcccctagaacactggtgctgctgctgtctggtgccctggctctgacacaaacatgggccggcagccacagcatgcggtact<br>ttttcaccagcgtgtccagacctggcagaggcgagcctagatttattgccgtgggctacgtggacgacacccagttcgtcagattcgattccgat<br>gccgccagccagcggatggaacctagagcaccttggatcgagcaagagggccccgagtattgggacggcgagacaagaaaagtgaaggccc<br>acagccagacacacagagtggatctgggaaccctgcggggctactacaatcagtctgaggccggctctcacaccgtgcagaggatgtacgga<br>tgtgacgtgggcagcgattggcggttcctgagaggctatcaccagtacgcctacgacggcaaggactatatcgccctgaaagaggatctgcggt<br>cttggacagccgccgatatggctgcccagaccacaaagcacaagtgggaagccgctcacgtggccgaacagctgagagcttatctggaagg<br>cacctgtgtggaatggctgcggagatacctggaaaacggcaaagagacactgcagcggacagacgcccctaagacacacatgacacacca<br>cgccgtgtccgaccacgaagccacacttagatgttgggccctgagcttctaccccgccgagatcacactgacatggcagagagacggcgagg<br>atcagacccaggataccgagctggtggaaacaagaccagccggcgacggcaccttccagaaatgggctgctgtggtggtgcctagcggccaa<br>gagcagagatacacctgtcacgtgcagcacgagggcctgcctaagcctcttacactgaggtgggagcccagcagccagcctacaatcccca<br>tcgtgggaatcattgccggcctggtgctgtttggcgccgtgattacaggtgcagtggtggccgctgttatgtggcggagaaagagcagcgacagaa<br>aaggcggcagctactctcaggccgccagctctgattctgcccagggctctgatgtgtctctgaccgcctgtaaagtc
BFP	gatccatgacgatatggtctcaaatttagcgactaaaatatcagttaacgctaaaatagagtagcctttatcgctgagaataatcgcatgtgct<br>cgtattcttactctatttttcggatggttagcaatcgcttctcttagggttgtttttcatagtctgttaaagtattaactgctttcattgtgatatagac<br>actgacgtaaaatactcattttaataccatattatcatatggtaaaggcagagaacggactctcgaaaacttattatcaactacttaacacta<br>atggttttaaaattagaaaacaacattatagcaatccacataaatatttggtcattcgtgcttgctaaaatcgattatagctgtgtcataatacc<br>aattccagtaattatctaaataactacttatctgaattggtattagtaagcatcctgcaataatttacagtaataatgtagctaaatttgtaataa<br>aatcattgacaaaaattaaattcgttataaataagcgaattaatatcaattatatatgtgtagttaaaattttaattcactattatatagagtcca<br>ttgtcatgaatcgagcaaacccaacattgaataactgattcataacaaatatttctctcttacttaaataaaaggtattctttatgtttaaaggta<br>atgttcaaggagtcggtacagttgaaaatatagataaaggagctaaatttcaaagccttcatggtgtttctttgttacccatagatgctgatctcc<br>aaagccacgatatcatttttccagaagatatattagagggagtgacttcaggagaattaatagcaattaacggagttcgtcttacagtggttcac<br>actgataaaagtatcgtacgttttgatataaatgatgcactagaactaaccaccttaggtcaattaaaggtaggggataaagttaatatagaga<br>aatcctttaaatttggagatatgactggaggtcgttcattatctggtattgtcaccggtgtcgcagatatcgttgaatttatagaaaaagaaaataa<br>tcgtcaaatatggattgaagctcctgaacacttaacggagtttctagtcgaaaaaaaatatatcggtgttgatggtgtttacttagttattgatgct<br>attgaaaataatcgcttttgcattaatttactcctagaaacagatatgaggtggtacaaaaaaggaagcaaggtgaatattgaaattccagata<br>ttgcaggtaattggtaaaatcagactgtaccgaaaagtttttctaataccaatacaattaattaaatgatctatttttacgcagaaaaatcgctt<br>aaaaataaggcaaaaattcttataagtagttattctacaatactaatttttaagtgatttaaccagtaagaatgaacagataattactggaattg<br>gtattacttcttttgaatcttcaatttttttgataaccatgctgaaaaaattatgatcacaaacataagcataaaattcgcgatagtttttattgtc<br>agcacaagaatactcctagaaggaagagcggctaacagctcgtaagtaccaaaacggaacaccaacatcaacgtaataatagaacaaaa<br>cgcaatcaaacgaccttttgttgtatttaatatagcctggtctttttcaacacaagcgcaagggttgttccccaacatgacgctgcacataca<br>aatattcatatg
TST tag	tggtcccatcctcagttcgagaaaggcggaggatctggcggtggaagcggtggatctgcttggagccatccacagtttgaaaagtga
GS linker(between construct and tag)	ggctctggtggctctggcggatctgctggtgga
