# Supplementary material for: An mRNA-based workflow validating neo-epitope presentation through HLA-I/peptide affinity purification
Source: Front Immunol. 2025 Jun 4;16:1566461. doi: 10.3389/fimmu.2025.1566461 (PMC12174155; doi:10.3389/fimmu.2025.1566461)
Supplement: Supplementary Figure 1 — Flow cytometry gating strategies. Gating strategy for (a) K562-cells and (b) StrepTactin beads. [file DataSheet1.pdf]

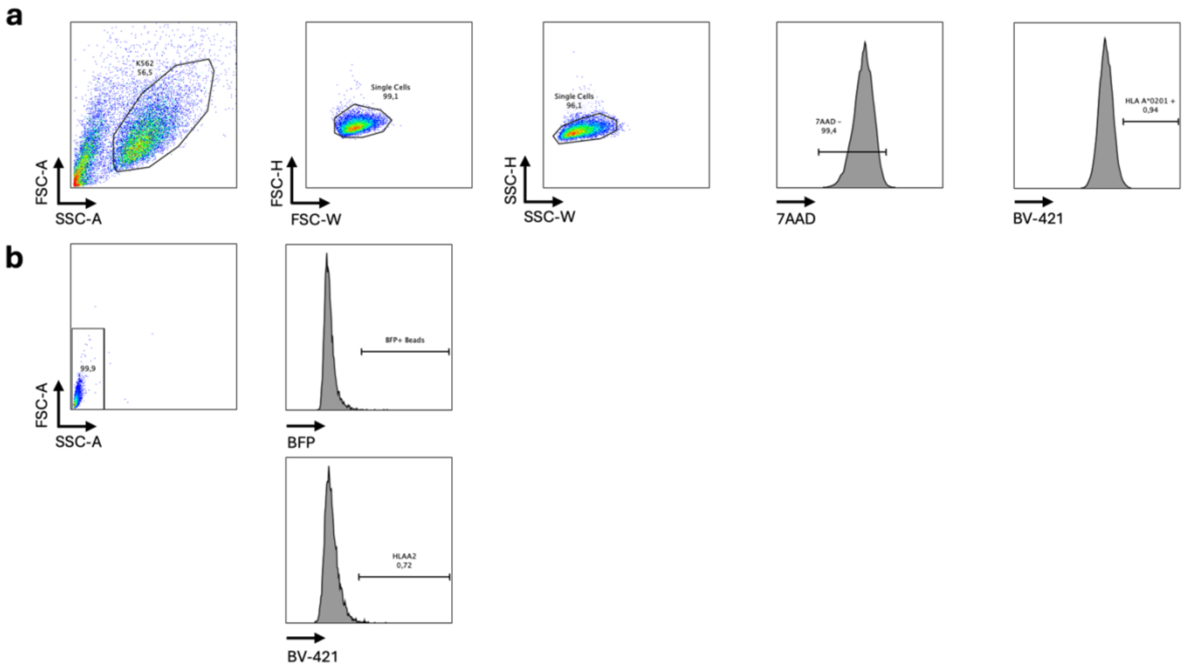

Figure S1: Flow cytometry gating strategies. Gating strategy for K562 cells (a). Gating strategy for streptactin beads (b).

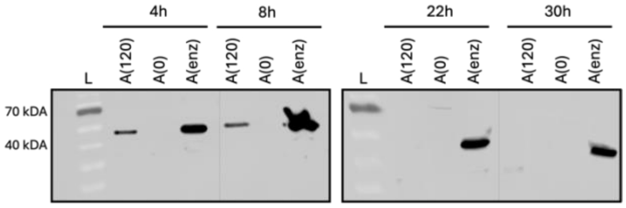

Figure S2: Western blot showing HLA-TST expression from mRNA generated with different poly-A tailing strategies, 4, 8, 22 and 30 hours after electroporation.

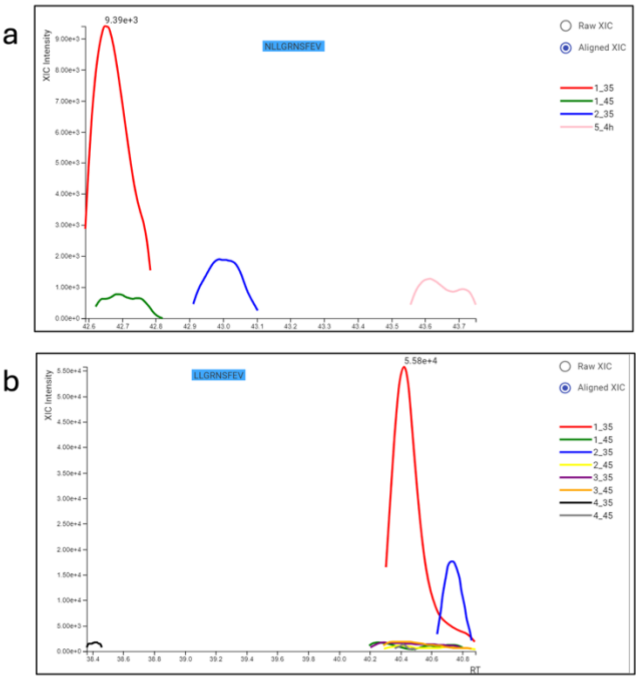

Figure S3: Chomatographs of the eluted peptide LLGNRSFEV for the TST-based approach(a) and the mAb-based approach (b).
